# Panel experts in shaping the nutrition and food safety guidelines for CYCWs in KwaZulu-Natal, SA

**DOI:** 10.4102/hsag.v30i0.2819

**Published:** 2025-06-16

**Authors:** Mumsy E. Chibe, Carin Napier, Heleen Grobbelaar

**Affiliations:** 1 Department of Life and Consumer Science, College of Agriculture and Environmental Sciences, University of South Africa, Johannesburg, South Africa; 2 School of Population Health, Faculty of Medical and Health Sciences, University of Auckland, Auckland, New Zealand; 3 Department of Food and Nutrition Consumer Sciences, Faculty of Applied Sciences, Durban University of Technology, Durban, South Africa

**Keywords:** child and youth care centres, development, food, handling, preparation, healthy eating

## Abstract

**Background:**

The study is based on the view that food contamination and limited knowledge of health and hygiene are some challenges Child and Youth Care Centres (CYCCs) face. The South Africa Nutrition Education Programme (NEP) was a conceptual framework within which the study is embedded. Despite the existence of relevant legislation and processes on food preparation, safety and healthy eating, South Africa’s CYCCs are still characterised by practice limitations.

**Aim:**

The study explores the use of expert panels in the review of newly developed guidelines for child and youth well-being.

**Setting:**

South African Experts in the field of CYCC and Academia.

**Methods:**

A qualitative approach was utilised to select three experts in Child and Youth Care, Child and Youth Care Education and Training and Food, Nutrition, and Community Research Education and Training. They were given 58 pages of guidelines to review and submit input. Thematic analysis was used to interpret the data.

**Results:**

The study’s findings included six main themes and related sub-themes. The main themes include nutrition terminology and words, healthy eating plans, menu planning, food safety and hygiene, food preparation and the layout of the guidelines. In general, the study findings can be highlighted as inclusion of low-cost, widely available, cost-effective meals, utilising SA-acceptable terminology and focussing on SA sources rather than global literature.

**Conclusion:**

The guidelines are critical because CYCCs in South Africa currently lack such guidelines. Furthermore, the expert reviewers acknowledged that the guidelines were applicable and necessary in South African child and youth care facilities.

**Contribution:**

Child and Youth Care Centres in South Africa will be able to incorporate the guidelines into their everyday food handling and preparation procedures.

## Introduction

The study aims to guide the development of child nutrition, food preparation, food safety and healthy eating guidelines using experts in the fields of academia, child and youth care (CYC), nutrition and food preparation. Literature depicts that the increasing vulnerability of children because of disease, conflicts and natural disasters led to a renewed interest in Child and Youth Care Centres (CYCCs) and workers (Mhizha & Nhedzi 2023). Most orphaned, abused, abandoned or destitute children are reported to end up in various CYCCs, where professionals are trusted with the responsibility of raising and meeting the children’s educational and developmental needs (Malatji & Dube 2017; Vision Child and Youth Care Centre 2019). This study was mainly informed by the findings of several studies. A study by Grobbelaar and Napier (2014) found that there is a need to improve the nutrition knowledge of Child and Youth Care Workers (CYCWs). Hansungule (2018) found that CYCWs were not effectively performing their duties, partly because of a lack of formal training. Molepo and Delport (2015) discovered numerous obstacles, including poor CYCW training and professional development. As a result, the purpose of this study was to develop, with the support of an expert panel, food preparation, food safety and healthy eating guidelines for CYCW in CYCCs in KwaZulu-Natal, South Africa.

## Background

The Constitution of the Republic of South Africa, 1996, protects children’s rights in general and the child justice system in particular (Reform & Tenants 1996). *The Children’s Act of 2005* assures the protection of children in need of care by establishing CYCCs to receive, create and safely care for children in accordance with *Chapter 10 of the Act* (Skelton & Courtenay 2015). Child and Youth Care Workers take on the responsibility to care for these children after they are placed in care in CYCCs. The Department of Social Welfare oversees CYCCs, which provide care for children and youth who are orphaned, abused, abandoned, homeless or victims of domestic violence. A CYCC is a care facility for children who do not live with their biological families (Jamieson 2013). In addition, section 193(2) of the *Children’s Act, 2005* (Act No. 38 of 2005) states that such a CYCC must be operated and maintained in accordance to the *Children’s Act, 2005*, and the structural, protection, health and other requirements of the community in the area where the CYCC is located (Rasool & Swart 2024). Regulations 75(1), 82 and 83 of the Act require CYCCs and staff to have the necessary qualifications and skills to operate and assist in the operation of the CYCC. While the Act does not specify specific qualifications, it guides the abilities required to lead to eligibility, such as advanced knowledge of child and youth work (Jamieson 2013).

A study by Dimba-Ndaleni, Motloung and Kasiram (2022) found that childcare workers are inexperienced and hence feel disempowered. Childhood and youth are life stages marked by physical, social, cognitive and behavioural diversity, so CYCWs must be properly educated about their roles and responsibilities, including involvement in the healthcare of at-risk children (Barford & Whelton 2010; Grobbelaar & Napier 2014). Meanwhile, a study in Finland by Swanzen and Jadrijevic (2014) compared the role of CYCWs to that of a parent, taking into account their intervention in assisting children with their well-being and other daily responsibilities. Mhizha (2020) and Hope and Van Wyk (2019) believed that children’s physiological development and maturity levels necessitated that CYCWs be prepared to keep them safe. Failure to practice appropriate nutrition and correct food handling, preparation and feeding procedures when caring for children can lead to foodborne infections and malnutrition (World Health Organization [WHO] 2017). This opinion is consistent with the WHO (2015) report, which emphasised that most foodborne infections are associated with food preparation using unsafe water, poor hygiene practices, inadequate food production and storage settings and low levels of literacy and education among CYCWs. Tanaka (2017:1) summed it up by stating that nutrition is a critical component of a child’s life. Caring for children also involves an understanding of the various stages of development and health, such as physical, social and emotional care and support (Tsao, Yeh & Lin 2024). As a result, CYCW functions must include nutrition, food safety, handling and preparation. This is supported by the fact that the *Children’s Act* broadly implies that a child is supported rather than abused or neglected, in accordance with the founding values of South African democracy, ‘Ubuntu’, which means dignity, equality and the advancement of human rights and freedom (Republic of South Africa 1996). However, the Act makes no mention of services such as nutrition, food safety, handling or preparation that CYCCs can use or follow whilst serving children and youth. As a result, the study’s goal was to bridge the gap by developing and validating child nutrition, food preparation, safety and healthy eating standards for CYCWs in CYCCs throughout South Africa using an expert panel.

### Rationale for the study

Physical, cognitive and emotional variables all have an impact on a child’s development. Growth and development require parents and carers to comprehend patterns and concepts (Ruffin 2019). An adequate diet, rich in foods from various food groups (carbohydrates, protein, fat, vitamins and minerals), is one of the healthy eating habits and principles required for a child’s maximum health, growth and development. Beliefs, attitudes and activities formed throughout infancy influence adult behaviour. There is growing evidence that health in childhood and adolescence impacts health in later life (Duncanson et al. 2019). However, countries around the world continue to struggle with various forms of malnutrition (WHO 2020b). Unsafe food puts global health at risk and harms everyone. Children and people with an underlying illness are most at risk, as the poor-quality food they eat promotes a vicious cycle of diarrhoea and malnutrition that jeopardises their nutritional status (WHO 2020b). Meanwhile, the literature reviewed indicated that care facility settings as explored by the study are not able to meet children’s basic needs, which primarily include adequate nutrition and safe food (Malatji & Dube 2017; Van Ijzendoorn et al. 2011). Some of the staff members do not clearly understand their positions and responsibilities regarding nutritional needs and safe foods provided to children and youth in CYCCs. There is no child nutrition, food preparation, food safety and healthy eating guidelines impacting CYCC operations and the ability to meet service delivery criteria (Hansungule 2018). There is also a lack of official or documented policies and processes for receiving, processing and serving food, as well as cleanliness and administration at CYCCs (Chibe, Napier & Grobbelaar 2024). Grobbelaar and Napier (2014) discovered that CYCWs had a limited awareness of relevant food safety and hygiene requirements. Most CYCWs were aware of how to properly handle fruits and vegetables; however, this was not the case in practice. For example, Chibe et al. (2024) discovered that fresh fruits and vegetables were not routinely washed before consumption. The results also showed that CYCWs lacked awareness of measures to prevent food contamination and had limited knowledge of the necessary washing processes for plates and utensils (Grobbelaar & Napier 2014).

### Conceptual framework

The Food and Agriculture Organization (FAO) and Nutrition Education Programmes (NEP) framework was adopted as the conceptual framework for this study and is not intended to be prescriptive but rather to stimulate conversation about the proper concerns, parameters, methodologies and processes for NEPs (Schmitt et al., 2019; Smith & Smitasiri 1997). Although the framework was specifically designed for a NEP, it was found to be relevant in other food practice-related programmes considering the factors that are covered within the framework. For this study, an expert panel was used in shaping Nutrition and Food Safety Guidelines for CYCCs in KwaZulu-Natal, South Africa.

## Research methods and design

A qualitative approach was utilised to develop guidelines for use by CYCCs. A selective sample strategy was employed to select three (*n* = 3) experts in CYC services, as well as food and nutrition. The respective experts were selected based on their diverse expertise, affiliations and work experience in the fields of CYC services and food and nutrition.

According to the research, expert judgements are becoming more prevalent in domains such as research, education and healthcare. Experts are characterised as knowledgeable individuals in a given discipline or field, who are usually arranged as a panel, individual members or both (Bruce, Langley & Tjale 2008). Prior to the development of the guidelines, a pilot study was undertaken at two CYCCs with (*n* = 30) CYCWs to assess the need for the guiding document. Findings from the pilot study were integrated into the final printed guidelines. To develop the guidelines, a literature review of books, journal articles and official reports on menu planning, nutritious recipes for children and adolescents aged 5–18 years, nutritional guidelines and safe food handling and preparation was carried out.

**FIGURE 1:**
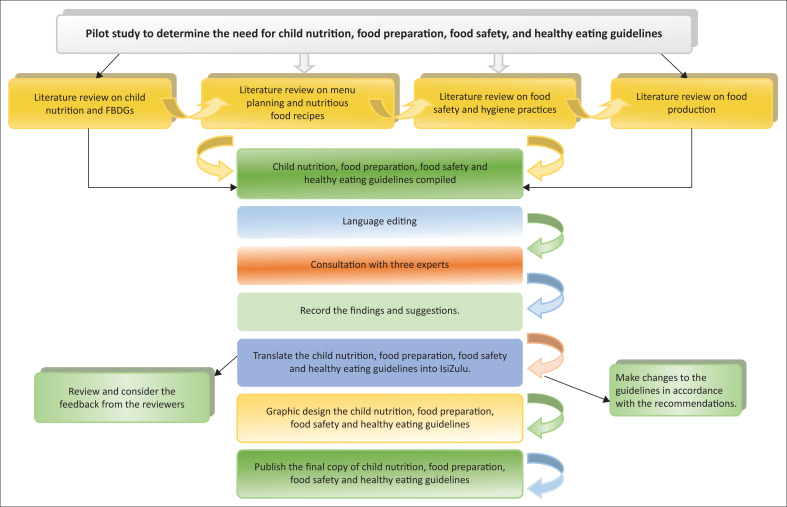
Framework for the development process of the child nutrition, food preparation, food safety and healthy eating guidelines.

Experts’ years of experience and of their contributions in food, nutrition and CYC, as well as their diverse backgrounds, affiliations and work experience were determined for the selection of the sample. To confirm the data’s credibility, specialists from various backgrounds were chosen from different provinces in South Africa. The researcher contacted all the experts via email. For confidentiality reasons, they are identified by codes, and their profiles are summarised as follows:

- Expert A: Nutrition and Research Methodology Lecturer and Supervisor, with 28 years of experience in institutions of higher learning.
- Expert B: Dietitian and Registered Nutritionist, with 35 years of experience as a strategic consultant in the CYC field.
- Expert C: Nutrition and Dietetics Educator with 33 years of experience in an institution of higher learning.

A 58-page completed and linguistically adjusted version of the Nutrition, Food Preparation, Safety and Healthy Eating Guidelines was distributed to experts in the format shown in Table 1, with the request to review and provide feedback on content accuracy, completeness and relevance. The experts were given 2 months to complete the task. The researchers then analysed the experts’ written comments and responses. The responses were collected and evaluated to identify reoccurring themes, concepts, patterns and commonalities, which were then coded.

**TABLE 1:** The structure of the developed guidelines.

Sections of the guidelines	Content covered
Section A: Nutrition and the South African Food-Based Dietary Guidelines	Nutrition and nutrients Description of the South African Food-Based Dietary Guidelines (FBDGs) Nutritional requirements Variety of food A variety of food on a plate Food consumption Benefits of a healthy eating plan South African FBDGs for healthy eating South African FBDGs and estimated portion sizes; enjoying a variety of foods; be active Make starchy foods part of your diet Eat plenty of fruits and vegetables every day Eat dry beans, split peas, lentils and soya regularly Have milk, maas or yoghurt every day Fish, chicken, lean meat or eggs can be eaten daily Drink lots of clean, safe water Use fats sparingly; choose vegetable oils rather than hard fats Use sugar and foods and drinks high in sugar sparingly
Section B: Menu Planning and Recipes	Menu planning Basic steps of menu planning Description of steps for menu planning Daily meal consumption pattern Suggested affordable healthy recipes Method and processes for standardising a recipe
Section C: Food safety and Hygiene Practices	The definition of hygiene The benefits of good hygiene Consequences of poor food hygiene Features of food preparation area Safety when preparing food Food service facility hygiene checklist Personal hygiene Wear protective clothing Procedure for keeping food safe during preparation Description of the food safety keys
Section D: Food Production	Standard procedures for food purchasing Food purchasing procedures The general process for receiving stock During receiving check for visible signs of food spoilage Food storage Use the following method to store food Procedure for storing frozen foods Procedure for storing refrigerated foods Issuing of food Food preparation Selection of a correctly coloured cutting board Food defrosting procedures Defrosting using a refrigerator Defrosting using water Defrosting using a microwave Cooking without defrosting Keeping food at a safe temperature after cooking Food serving procedures After food preparation Treatment of leftovers Treatment of food waste Dishwashing techniques

### Data analysis

Thematic analysis was employed to analyse the data gathered from expert feedback. The data were reviewed, themes emerged and conclusions were reported utilising Braun and Clarke’s (2022) six-step theme analysis process, which included familiarisation, generating initial codes, searching, reviewing, defining and naming themes and producing the report.

Furthermore, the primary researcher performed the analytic procedure. To assure the authenticity of data gathering and analysis, co-researchers verified data audit trails, and member checks were carried out. The audit trail procedure is quite useful for validating the conclusions of a qualitative study (Carcary 2009). Member checking, on the other hand, helps to maximise the study’s trustworthiness by presenting data transcripts and soliciting feedback from participants (McKim 2023). In the first case, the two co-researchers confirmed full adherence to the structure and procedure used in data collection and analysis, including coding. In the second case, a member check was performed to confirm that the researcher accurately and sufficiently recorded the participants’ statements, views and experiences.

### Ethical considerations

This article is based on the doctoral study titled: Development of nutrition, healthy eating and food preparation guidelines for Child and Youth Care Centres in KwaZulu-Natal, South Africa. The ethical clearance to conduct the study was obtained from the Durban University of Technology (DUT) Institutional Research Ethics Committee (IREC No: 076/15). Experts were sought through email. A written email certifying that they consent to participate was obtained. Anonymity was preserved by not publishing the names and addresses of the experts in all activities and the final report; codes were utilised during the reporting process.

## Results and discussion

This section presents the findings in which the experts provided their comments and responses about the guidelines’ content. The experts’ responses showed remarkable overlap. The combined responses are presented. Five themes emerged from the data, each with several sub-themes. Each of these themes and sub-themes is summarised in Table 2.

**TABLE 2:** Themes and sub-themes by the expert panel.

Themes	Sub-themes
1. Nutrition terminology and words	-
2. Healthy eating plan	-
3. Menu planning	3.1. Use of low-cost food
4. Food hygiene and safety	4.1. Adoption of WHO Five Keys to Safer Foods 4.2. Use of artistic illustrations
5. Food preparation	5.1. CYCC infrastructure
6. Layout of the guidelines	6.1. Relevance 6.2. Layout 6.3. Issues of copyrights for Internet pictures

CYCC, Child and Youth Care Centre; WHO, World Health Organization.

### Theme 1: Nutrition terminology and words

This theme emerged when the experts reviewed the nutritional terms or words used in the guidelines. It became apparent that understanding and knowing the correct nutrition terms may make it easier for a person to make better food choices. For instance, one of the experts B stated that words that can be understood by the target audience should be used:

‘Nutritious meals or a balanced diet terms should be replaced with evidence-based words that are understandable to the target audience, for example, a statement that reads as follows: meal or diet requirements, a balanced, safe eating plan or trend, and a good mixed meal.’ (Expert A)

Meanwhile another participant stated:

‘[*T*]hat basic, easy-to-understand terms. The words used are extremely difficult to explain and recall.’ (Expert C)

According to the literature, nutrition terminologies should be understandable to the general audience (Buckton, Lean & Combet 2015); however, not much research has been undertaken to investigate the consistency and accuracy of their use (Matthews, Palmer & Capra 2018).

### Theme 2: Healthy eating plan

Serving healthier foods is a vital step towards improving one’s diet. However, planning and preparing nutritious, balanced meals is challenging and time consuming (Sufahani & Ismail 2014). The issue of healthy eating emerged in the reports presented by the experts, and Expert A described their opinions by expressing that:

‘The content of the guidelines looks good and relevant, but I suggest that you add some information on healthy eating plan for children.’ (Expert A)

Whereas eB on this theme supported eA by narrating:

‘[*T*]hat health eating information should include some literature, artwork, sketches, or images.’ (Expert B)

The expert panel’s views are congruent with Sanjana, Konjengbam and Christina (2023), who said that healthy eating habits must be established during childhood. Nourishing, well-balanced diets are critical for optimal growth, immunity, physical and mental development, health and well-being and a lower chance of chronic disease later in life. Common nutrition-related concerns in young people include anaemia, growth retardation, eating disorders and obesity.

### Theme 3: Menu planning

This theme developed from the expert panel’s assessment of the menu planning process is presented in the guidelines. It was agreed that menu planning is crucial to successfully providing services to children and youth residing in CYCCs. For example, one reiterated the following narrative:

‘Include low-cost menus that are also showing portion sizes to be served for different ages, use realistic example with times for meals and snacks.’ (Expert B)

Participant two’s narrative was supported by eA, who stated to ‘use food items that are accessible and suitable for children and young people’.

According to research, menus in childcare settings should be designed to provide nutritious meals and snacks necessary for the young children’s optimum health and growth. Furthermore, menus should be developed in a creative and enticing manner that can help to increase the adoption of new foods and encourage lifetime healthy eating habits. Menus must also strike a balance between the requirements and desires of the children in their care, the expectations, the constraints of the setting’s space, time and budget and the intricate nuances of government policies and regulations (Mann 2024).

### Theme 4: Food hygiene and safety

The review revealed a trend on food hygiene, emphasising the usage of the WHO Five Keys (WHO 2006) as a reference guide intended to educate all food handlers on safe food handling practices to address food hygiene and safety practices. Expert 1 expressed her opinion by echoing the following statement:

‘Align the guidelines with the WHO Five Keys to Safer foods to make them more relevant.’ (Expert A)

Additionally, another expert stated to:

‘[*U*]se photos which some can be obtained from Department of health to illustrate and display distinct concepts of food hygiene.’ (Expert B)

The experts’ views are consistent with those of Al Bayari et al. (2023), who stated that foodborne illness outbreaks are typically caused by improper food handling practices by food handlers, such as general unsafe food handling practices, cross-contamination and insufficient temperature control (Al Bayari et al. 2023). Meanwhile, a lack of food safety knowledge and practices can increase the incidence of foodborne infections (Islam et al. 2023).

### Theme 5: Food preparation

Food preparation refers to the steps taken to ensure that food is safe for consumption and has the desired flavour. The assembly process includes gathering raw foods and preparing the food for consumption. It also includes any portioning, packaging, assembly and processing that changes the form of food (Helfer 2024). A theme on proper food preparation procedures in a correctly designated kitchen is important. Experts C and B narrated the following statement:

‘Be clear on the procedure for preparing food that include different food groups [*starch, protein, vegetable and salad*] and portion sizes for different age group.’ (Expert C)

‘Be clear about who can utilise the guidelines: are they designed for CYCCs with modest and large scales?’ (Expert B)

### Theme 6: Layout of the guidelines

The guidelines should include some words and pictures for the target audience to be able to understand it easier. The theme on the layout of the guidelines was identified in all the reviews. Experts B and Expert B revealed the following narrative:

‘I suggest that you add some artwork, sketches, or images that correspond to the subject should be included to make the guideline to be easier to follow and understand the content.’(Expert A)

‘Images obtained from the internet must be copyright free and from an open source.’ (Expert B)

The expert’s opinions were consistent with those of Veiga-Díaz (2023), who noted that artwork and graphics have been identified in studies as a significant tool in education for attracting attention, improving clarity, creating a code, marking objects and distinguishing items.

## Conclusion

The results show that using experts had a favourable impact on the study’s outcomes. Experts provided valuable insights on nutrition terminology, healthy eating plans, menu planning, food safety and hygiene, food preparation and recommendations layout. It also highlights the importance of using simple and understood nutrition terms, such as healthy eating plans for children, choosing accessible and appropriate food items for menu planning, complying with the WHO food safety criteria and following clear food preparation practices. It also recommends incorporating artwork, sketches and visuals to help readers understand the content. As a result, the developed guidelines were approved as a valid learning resource for CYC settings. The experts further stated that, with adequate modifications and implementation of the recommendations, it would be feasible to reach the intended audience effectively.
